# Impact of Simulated Human Gastrointestinal Digestion on the Functional Properties of Dietary Fibres Obtained from Broccoli Leaves, Grape Stems, Pomegranate and Tomato Peels

**DOI:** 10.3390/foods13132011

**Published:** 2024-06-25

**Authors:** María Ángeles Rivas, Santiago Ruiz-Moyano, María Vázquez-Hernández, María José Benito, Rocío Casquete, María de Guía Córdoba, Alberto Martín

**Affiliations:** 1 Departamento de Producción Animal y Ciencia de los Alimentos, Nutrición y Bromatología, Escuela de Ingenierías Agrarias, Universidad de Extremadura, Avda. Adolfo Suárez s/n, 06007 Badajoz, Spain; mrivasm@unex.es (M.Á.R.); m.vazquez.hern@gmail.com (M.V.-H.); mjbenito@unex.es (M.J.B.); rociocp@unex.es (R.C.); mdeguia@unex.es (M.d.G.C.); amartin@unex.es (A.M.); 2 Instituto Universitario de Investigación de Recursos Agrarios (INURA), Universidad de Extremadura, Avda. de la Investigación s/n, 06006 Badajoz, Spain

**Keywords:** digestion process, dietary fibre, by-products, antioxidant capacity, antiproliferative activity

## Abstract

This study aimed to analyse the impact of a simulated human digestion process on the composition and functional properties of dietary fibres derived from pomegranate-peel, tomato-peel, broccoli-stem and grape-stem by-products. For this purpose, a computer-controlled simulated digestion system consisting of three bioreactors (simulating the stomach, small intestine and colon) was utilised. Non-extractable phenols associated with dietary fibre and their influence on antioxidant capacity and antiproliferative activity were investigated throughout the simulated digestive phases. Additionally, the modifications in oligosaccharide composition, the microbiological population and short-chain fatty acids produced within the digestion media were examined. The type and composition of each dietary fibre significantly influenced its functional properties and behaviour during intestinal transit. Notably, the dietary fibre from the pomegranate peel retained its high phenol content throughout colon digestion, potentially enhancing intestinal health due to its strong antioxidant activity. Similarly, the dietary fibre from broccoli stems and pomegranate peel demonstrated anti-proliferative effects in both the small and the large intestines, prompting significant modifications in colonic microbiology. Moreover, these fibre types promoted the growth of bifidobacteria over lactic acid bacteria. Thus, these results suggest that the dietary fibre from pomegranate peel seems to be a promising functional food ingredient for improving human health.

## 1. Introduction

Today, agricultural industries generate large amounts of agricultural by-products throughout the world. Agricultural by-products consist of waste, inedible parts derived from the cultivation and processing of food products [1]. The agricultural residues of crops are composed of leaves, flowers, stems and roots, while the by-products of the food industry include fruits, peels and discarded seeds [2]. The generation of agricultural residues and by-products causes a serious economic and environmental problem [3]. However, they may contain a high concentration of valuable bioactive compounds, such as phenolic compounds [4,5,6], terpenes [7], fatty acids [8,9], polysaccharides [10,11] and proteins [12]. In addition, it should be noted that plant by-products are a powerful source of dietary fibres [13,14,15], which have various functional properties and are associated with applications in the food industry as well as many benefits for human health [16]. Therefore, their extraction and application for different purposes in various fields, such as the food, pharmaceutical, cosmetic, textile and biofuel industries, can result in new value-added products [17,18].

The physiological effects of the by-products of dietary fibre depend on the composition and structural characteristics of the plant cell wall, as well as its techno-functional properties [19]. Previous studies reported that tomato peel, composed of approximately 48% insoluble dietary fibre and 9% soluble dietary fibre [20], is an important source of high-quality dietary fibre [21] and has excellent functional properties, in particular, a high gelling capacity [22] and a high capacity for glucose retention, which could have a protective effect against postprandial hyperglycaemia [23]. In addition, other vegetable by-products, such as grape stems, pomegranate peels and broccoli leaves, showed good functional and technological properties. The peel of the pomegranate was found to be rich in antioxidant dietary fibre [24], while the fibre from grape stems [15,25] and broccoli leaves [26,27] was shown to have a prebiotic capacity and the capacity to stimulate an increase in the amount of short-chain fatty acid (SCFA) in vitro, respectively.

Most previous works on isolated fractions or extracts of dietary fibre from various by-products focused on evaluating their composition, structure and functional properties; however, few studies analysed their functional properties throughout the simulated digestion process. Ribeiro et al. [28] studied the impact of in vitro simulation of gastrointestinal (GI) digestion on antioxidant dietary fibre powder from olive pomace. They concluded that dietary fibre, containing free and bound phenolics, can reach the colon and potentially offer health benefits, such as antioxidant, antimicrobial and anti-inflammatory activities. Additionally, dietary fibre may positively interact with lipids by decreasing the bioaccessibility of saturated fatty acids and facilitating the absorption of unsaturated fatty acids. To understand the potential impact of dietary fibre on human health when incorporated as a food ingredient, it is essential to study the effects of GI stresses on its structural and functional properties. Thus, the objective of this study was to evaluate, using a computer-controlled GI digestion simulator, in vitro digestibility, focusing on identifying the composition, structure and functional properties of extracts of dietary fibre from pomegranate peel, tomato peel, broccoli stems and grape stems. This research can serve as a scientific basis for promoting the use of dietary fibre as a functional food and/or additive in the food industry of agricultural by-products that have, to date, been undervalued.

## 2. Materials and Methods

### 2.1. Plant Material and Dietary Fibre Extraction

The by-products used in this study (pomegranate peels, tomato peels, broccoli leaves and grape stems) were provided by industries from the Autonomous Community of Extremadura, Spain. They were dried to a moisture content of approximately 6% in a forced air oven (Model Digitronic-TFT, SELECTA, Barcelona, Spain) with a flow rate of 2 m^3^/min and an air temperature of 45 °C, followed by vacuum packaging individually in a plastic bag using a vacuum packaging machine (Model SV-420S, Sammic, Azkoitia, Spain). The vacuum bags were stored at room temperature until use.

The extraction of dietary fibre from the by-products was carried out following the alcohol-insoluble residues (AIR) method described by Rivas et al. [15]. Once extracted, the extracts were ground and passed through a 1 mm sieve.

### 2.2. Preparation of a Base Feed Supplemented with Different Types of Dietary Fibre

Chickpea paste, obtained from washed, cooked chickpeas, combined with 400 mL of sterile distilled water and ground in a Thermomix (Wuppertal, Vorwerk, Germany) at high speed for 5 min, was used as a dietary fibre control. Dietary fibre extracts (50 g) from each by-product were individually added to the chickpea paste by normalising the amount of fibre (to approximately 20% of the dried extract) by adding water.

### 2.3. Faecal Inoculum

A faecal sample was collected from a 45-year-old, healthy human volunteer, who was on a non-specific Mediterranean diet, had no metabolic or GI diseases, did not smoke and had not received any antibiotics or pre- or probiotic supplements for at least 6 months before the faecal donation. Voluntary informed consent was obtained from the donor prior to this study. A 20 g faecal sample was diluted in 80 mL of 58% glycerol in phosphate-buffered saline (PBS) 0.1 M at pH 7.0. Immediately afterwards, the faecal inoculum was stored at −80 °C until use. Only one donor was used for the experiment to prevent diversity associated with the use of different faecal samples.

### 2.4. Human Simulated Digestion: Experimental Design

For the simulated digestion of the different dietary fibres and the control, a dynamic in vitro simulator model of the human digestive system, consisting of three-bioreactor BIOSTAT A systems (Sartorius Stedim Biotech, Göttingen, Germany) connected in series by peristaltic pumps, was used. These three glass vessels simulated the conditions of the stomach, small intestine and colon (ascending, transverse and descending colon), respectively. The parameters of simulated digestion in each section of the system were entirely computer-controlled and are shown in Table 1. The initial feed consisted of 750 mL of sterile PBS for each bioreactor, which was equilibrated at specific initial conditions of temperature, pH and O_2_ pressure for 2 h. Before the start of the digestion process, the 3rd bioreactor (colon compartment) was inoculated with 25 mL of De Man Rogosa Sharp broth (MRS; Condalab, Madrid, Spain) medium and 10 mL of faecal inoculum, so a 12 h stabilisation phase was necessary for the colon microbiome before this bioreactor could be used effectively. During the digestion process, peristaltic pumps added a specific amount of food supplements, HCl, pepsin, NaHCO_3_ and pancreatic and biliary fluids (Sigma-Aldrich Chemistry, St. Louis, MO, USA) to simulate the stomach and small intestine (Table 1). The digestion process was repeated twice for each dietary fibre and control, and samples were taken for analysis in triplicate at different times during the process. Samples were collected at the initial phase, end of the stomach stage (sampling 1_1 and 1_2), end of the small intestine stage (sampling 2_1 and 2_2) and colon stage: 8 h (ascending: sampling 3_2), 20 h (transverse: sampling 3_3) and 36 h (descending: sampling 3_4). 

### 2.5. Characterisation of Digestion Extracts 

#### 2.5.1. Dietary Fibre Content

The dietary fibre content of the different by-products was calculated before and during the different stages of the digestion process by the AIR method described in Section 2.1. The results were expressed in grams of AIR/100 g of solid residue. 

#### 2.5.2. Dietary Fibre Composition

To determine the content of galacturonic acid and the profile of neutral sugars, the dietary fibre extract underwent hydrolysis with 12 M sulfuric acid (3 h at room temperature and 1 h at 100 °C). Subsequently, the released monosaccharides and galacturonic acids were determined using HPLC. The HPLC analyses in this study were conducted using an Agilent LC 1260 Infinity II HPLC system (Waters, Milford, MA, USA), comprising a separation module and an RI detector. The HPLC system was equipped with a Rezex ROA-Organic Acid H+ (8%) column (7.8 mm ID × 150 mm; Phenomenex, Torrance, CA, USA). In isocratic mode, the mobile phase consisted of water with a flow rate of 0.6 mL/min. In elution mode, the sample injection volume was 10 μL, the column temperature was 80 °C and the detector temperature was 40 °C.

#### 2.5.3. Non-Extractable Phenolic Compounds and Their Antioxidant Capacity

The determination of non-extractable phenolic compounds in dietary fibre and their antioxidant capacity involved an initial extraction of phenolic compounds from the AIRs, following the method outlined by Rivas et al. [15]. 

The phenolic content in the extracts was quantified using the Folin–Ciocalteu reagent [29] via a UV-1800 spectrophotometer (Shimadzu Scientific Instruments, Columbia, MD, USA), with gallic acid as the standard. The results are expressed as milligrams of gallic acid equivalents (GAE) per 100 g extract.

The antioxidant activity of the extract solutions (10 mg of extract per millilitre of ethanol) was assessed by bleaching the violet solution of the 1,1-diphenyl-2-picrylhydrazyl radical using the DPPH method, as described by Teixeira et al. [30], and by measuring the ability to eliminate the 2,2′-azinobis (3-ethylbenzothiazoline-6-sulfonic acid) radical (ABTS) following the method of Re et al. [31]. Total antioxidant activity was expressed as milligrams of Trolox per 100 g of extract.

#### 2.5.4. Microbial Population

For microbial counts, 10 mL of each sample from each stage of digestion was placed aseptically in a sterile plastic bag with 90 mL of 1% peptone water (Condalab) and homogenised for 120 s in a stomacher instrument (Lab-Blender 400 Seward Lab., London, UK). Serial 10-fold dilutions were made with peptone water, and 0.1 mL aliquots of each dilution were inoculated onto agar plates. Total viable bacteria were counted on plate count agar (PCA; Condalab) after incubation at 30 °C for 48 h. Lactic acid bacteria (LAB) were enumerated on MRS agar (Condalab) acidified to pH 5.6 with acetic acid (10%) at 30 °C after 48 h. Bifidobacteria were enumerated on MRS agar (Condalab), supplemented after sterilisation with L-cysteine-HCl 500 mg/L, Mupirocin 100 mg/L, Kanamycin 25 mg/L, 2,3,5-triphenyltetrazolium chloride 25 mg/L, Polymyxin B 4.28 mg/L [32], under anaerobic conditions, using an Oxoid AnaeroGen 3.5 L sachet (Thermo Scientific, Waltham, MA, USA) and an anaerobic jar, for 72 h at 37 °C. Enterococci were counted on Slanetz and Bartley agar (SB; Condalab) at 37 °C for 48 h. The staphylococci were counted in Baird-Parker agar (BP; Condalab), supplemented with potassium tellurite and egg yolk emulsion after incubation at 37 °C for 48 h. Enterobacteria were counted on violet red bile glucose agar (VRBG; Condalab) after incubation at 30 °C for 24 h. Yeast was counted on potato dextrose agar (PDA; Condalab), acidified to pH 3.5 with a sterilised solution of tartaric acid (10%) after incubation at 25 °C for 72 h. Microbial counts were expressed in log CFU/mL.

#### 2.5.5. SCFAs

To assess the impact of each dietary fibre on microbial activity, the level of SCFAs across various stages of digestion was measured. Five hundred microlitres of the digested extract was combined with 500 µL of ultrapure water and 100 µL of internal standard (2-ethylbutyric acid). Subsequently, 0.5 µL of this mixture was injected into a gas chromatograph equipped with a split/split-less injector and a flame ionisation detector (Shimadzu 2010 Plus). SCFAs were separated using a DB-FFAP capillary column (30 m × 0.25 mm id; 0.25 µm) using the chromatography conditions described by Rivas et al. [26]. Identification of individual SCFAs was achieved by comparing their retention times with those of the reference standard mixtures (acetic acid, propionic acid, butyric acid, isobutyric acid, valeric acid, isovaleric acid and hexanoic acid; Sigma-Aldrich). SCFA concentrations were determined as the ratio of the peak area of the analyte to that of the internal standard (2-ethylbutyric acid), according to [33].

#### 2.5.6. Soluble Oligosaccharides 

An HPLC analysis was performed using an Agilent 1260 Infinity LC system (Agilent Technologies) equipped with a refractive index detector (RID). Data acquisition was controlled by OpenLAB CDS ChemStation Edition™ software (Rev. C.01.10) (Agilent Technologies). Prior to injection, a 500 μL aliquot of the digestion medium was diluted in 1.0 mL of HPLC water and filtered through a 0.45 μm nylon membrane. An injection volume of 10 μL was used. Chromatographic separation was performed using a Phenomenex Rezex RNO-Oligosaccharide Na+ (4%) column (200 × 10 mm ID; Phenomenex) with a particle size of 12 μm and a Rezex RNO-Oligosaccharide Na+ (4%) guard cartridge (60 × 10 mm ID; Phenomenex) with 8.0 μm internal particles, protected by a PL Hi-Plex H guard column (60 × 10 mm; Rezex TM RNO-oligosaccharide Na+). The column temperature was maintained at 80 °C, while the RID flow cell temperature was set to 40 °C. The mobile phase consisted of HPLC water with a flow rate of 0.5 mL/min and a run time of 20 min. Detection employed the RID to measure changes in the refractive index. 

#### 2.5.7. Antiproliferative Activity

The antiproliferative test was performed using the human colorectal adenocarcinoma cell line HT-29 obtained from the America Type Culture Collection (ATCC^®^ HTB-38; Manassa, VA, USA). The cells were seeded at a density of 104 cells per well in 96-well flat plates in high-glucose, glutamine-free, Dulbecco’s Modified Eagle’s Medium (DMEM; Gibco-Thermo Fisher Scientific, Waltham, MA, USA) containing pyruvate, supplemented with 10% heat-inactivated foetal bovine serum (FBS; Gibco-Thermo Fisher Scientific), 1% L-glutamine (200 mM; Gibco-Thermo Fisher Scientific) and antibiotics, including 100 IU/mL penicillin and 100 μg/mL streptomycin (Gibco-Thermo Fisher Scientific). After incubation for 24 h at 37 °C in an atmosphere containing 5% CO_2_, culture media were substituted with 200 µL of complete culture media, supplemented with 50 µL of digestion extracts sterilised by filtration through 0.22-μm filters (Thermo Fisher Scientific). The extracts corresponding to the initial phase were previously diluted 1 in 8, those from the small intestine were diluted 1 in 2 and those from the colon were added without dilution. After 24 h of treatment, the culture medium was removed, and the cells were treated with 3-(4,5-dimethylthiazol-2-yl)-2,5-diphenyltetrazolium solution (MTT, 0.5 mg/mL; Sigma-Aldrich) for 1 h. The formazan blue crystals were then dissolved in 200 μL of dimethyl sulfoxide (DMSO; Sigma-Aldrich), and the absorbance was measured at 570 nm using a Fluostar Optima microplate reader (BMG LABTECH, Offenburg, Germany). Cells treated with hydrogen peroxide (2 mL/100 mL) were used as the positive control, while untreated cells were used as a negative control. The antiproliferative effect was calculated as the percentage growth inhibition relative to that of negative control cells.

### 2.6. Statistical Analysis

Data were analysed using SPSS for Windows (version 21.0, IBM Corp.) and descriptive statistics were calculated. Differences within and between groups (dietary fibre type) in stomach and small intestine simulations were assessed by a one-way analysis of variance (ANOVA), followed by Tukey’s HSD test (*p* ≤ 0.05) for between-group comparisons. For colon simulation samples, a two-way ANOVA was employed, using ‘dietary fibre type’ and ‘colon stage’ as inter- and intra-subject factors, respectively. Additionally, principal component analysis (PCA) was performed on the correlation matrix of the variables.

## 3. Results and Discussion

### 3.1. Content and Composition of the Dietary Fibre in Digestion Extracts

Table 2 shows the initial dietary fibre parameters of the control (chickpea) and by-products analysed. The dietary fibre content ranged from 188 g/kg for tomato peel to 227 g/kg for chickpea (control), without significant differences. The dietary fibre values of the chickpea agree with those published by other authors [34]. Concerning the dietary fibre constituents, statistically significant differences were found in all reducing sugars analysed. Specifically, broccoli leaves and grape stems presented lower glucose values than the control, whereas these by-products and the pomegranate peel presented the highest values of xylose, galactose and mannose compared to the control and the tomato peel. Rivas et al. [26] characterised the dietary fibre obtained from several vegetable by-products to study the improvement of the viability and metabolism of intestinal probiotic bacteria, obtaining similar results to ours for the composition of neutral sugars for winemaking by-products, broccoli, tomato and pomegranate peels. The higher glucose values for the tomato and pomegranate peels can be explained by the high cellulose content present in fruit and vegetable skins and peels [35]. No significant differences were found in the galacturonic acid and rhamnose/arabinose content of the dietary fibres from the by-products under study (Table 2).

During the simulated digestion process, after passage through the stomach, a dilution effect on the fibre content was observed for all dietary fibres. It should be noted that in the case of broccoli leaf and pomegranate digestions, this reduction was significantly higher than that of the others (Table 2 and Table 3). Although most dietary fibres cannot be digested under simulated saliva and stomach conditions [36], part of the soluble dietary fibre disappeared in the stomach with the solubilisation of non-cellulosic and non-starch polysaccharides [37]. The composition profiles of dietary fibre, in general, were not affected after passage through the stomach, as expected from the published works of other authors [38,39].

In the case of the small intestine, the dietary fibre values decreased due to the dilution effect associated with the change of the bioreactor, pH adjustment and the addition of a pancreatin and bile salt solution (Table 1). The overall differences in dietary fibre digestion found in the stomach stage for both total dietary fibre and its constituents were maintained in the small intestine simulation (Table 3). However, lower values of glucose and higher values of xylose, galactose and mannose were observed in the dietary fibre constituents with respect to the previous digestion stage. The higher values of neutral sugars (mainly xylose, galactose and mannose) with respect to the stomach stage, associated with a high content of galacturonic acid in the samples, seem to indicate a greater degradation of pectin. The dietary fibre with shorter chains and branches, as well as a loosening of the structure, allows better hydrolysis of the polysaccharides studied during the analysis and, finally, greater detection of these monosaccharides [40].

The dietary fibre values in the stages of colon digestion showed low fermentation (Table 3), which is positively associated with an impact on gut health [41]. Finally, no relevant changes were observed in the differences between the dietary fibre from the control and by-products with respect to the small intestine. The most easily fermentable substrates are characterised not only by their chemical composition but also by their ease of access by the microbiota [42]. In terms of dietary fibre composition, no significant differences were noted among the digestions in the galacturonic acid content. There appears to be a slight reduction in the monosaccharide content compared to the previous stage. Additionally, when comparing across colon stages, values consistently show lower levels in the samples from the distal colon. These findings are anticipated, as microorganisms tend to metabolise monosaccharides more rapidly than carbohydrates with longer chains, making them the preferred fermentation substrates. This observation aligns with the research by Stewart et al. [43], which emphasised the significance of the carbohydrate chain length in fermentation. They highlighted the rapid fermentation of short-chain fructans, primarily occurring in the proximal colon.

### 3.2. Functional Properties of Dietary Fibre

The non-extractable phenols associated with dietary fibre are shown in Table 3. The initial highest value was found in the pomegranate peel, followed by the grape stems, whereas the broccoli leaves, tomato peel and the control presented the lowest values. Previous studies corroborate the high values of non-extractable phenolic compounds found in these by-products [15,26]. The high initial antioxidant capacity of the pomegranate peel and grape-stem digestions, evaluated by both ABTS and DPPH methods, evidenced the positive relationship between the content of non-extractable phenols and the antioxidant capacity of these dietary fibres [44,45]. However, antioxidant capacity has also been associated with other residual compounds linked to dietary fibre, such as some soluble polysaccharides [46], terpenoids [47] and fatty acids [48], among others.

During the simulated digestion process, after passage through the stomach, the losses of phenolic compounds associated with dietary fibre are relatively small, since the dietary fibre protects them from GI conditions [49,50]. However, the decreasing tendency in the values of non-extractable phenols may be due to the degradation or loosening of the dietary fibre structures under digestion conditions [51], a process that can favour antioxidant activity due to greater accessibility to the linked compounds that support this activity (Table 3). In fact, the antioxidant capacity of polyphenols depends not only on the quantity but also on the percentage that can be released under intestinal tract conditions and therefore remains available for absorption [52]. As in the initial stage, the fibre added from pomegranate peel and grape stems showed the highest antioxidant activity in the simulated stomach stage.

At the end of the small intestine stage, the content of non-extractable phenolic compounds associated with dietary fibre tended to decrease in the broccoli leaf and grape stem digestions, whereas it increased in the rest of the dietary fibres tested, especially in the pomegranate peel (Table 3). This result agrees with the DPPH values observed in pomegranate dietary fibre digestion. The increase in non-extractable phenolic compounds may be because the phenolic acids released during gastric processing are reabsorbed into the cell wall under the small intestinal conditions [53]. In any case, for all dietary fibres evaluated, the antioxidant activity values were higher than those obtained at the beginning of digestion, demonstrating that dietary fibre protects the compounds responsible for antioxidant activity in its passage through the GI tract [54]. The depolymerisation of polysaccharides and changes in reducing sugars after simulated digestion may influence antioxidant activity [55,56].

Finally, approximately 50% of the initial phenolic content found in the broccoli leaf, pomegranate-peel and grape-stem dietary fibres reached the colon (Table 3). For control and tomato peel digestions, the content of polyphenols associated with the fibre was similar or higher in the colon stages compared to the initial contents. In general, the results of the antioxidant capacity, ABTS and DDPH data suggest that there was no substantial loss of this capacity after digestion and that it even increased in some cases. These data suggest that the phenolic compounds associated with dietary fibre are not completely bioaccessible in the small intestine, so a significant fraction moves to the colon, where it could act as a substrate for microbiota modulation and may have a positive impact on health. Indeed, N-EPC and DPPH decreased in the distal colon stage compared to the proximal colon stage. In this context, the dietary fibre extracted from the pomegranate peel stood out from the rest of the fibres studied. 

Regarding the results of antiproliferative activity, no significant differences in the initial stage were found among the different dietary fibres, with the values of cell survival oscillating between 80.4% and 103.2% compared to the positive control (Table 2). The values of the antiproliferative capacity of dietary fibre also did not show significant differences after passage through the stomach (Table 3). However, after passing the small intestine and colon stages, overall, the digestion of the dietary fibre from the broccoli leaves, pomegranate peel and grape stems showed a greater antiproliferative capacity of HT-29 cells compared to the control. These results agree with those published by other authors. Sharma et al. [57] demonstrated in their study that the xyloligosaccharide-rich dietary fibre from Azadirachta sawdust inhibits the growth of human colorectal cancer (HT-29) cells.

### 3.3. Oligosaccharides

The amounts of oligosaccharides (degree of polymerisation: 2–7) present in the dietary fibres studied in the simulated stomach and small intestine digestion stages are shown in Table 4. The pomegranate peel presented the highest value of total oligosaccharides (38.71 g/L) in the simulated stomach, followed by the tomato peel and grape stems, with 29.87 and 17.58 g/L, respectively. These differences were mainly associated with DP7, which was the predominant oligosaccharide found in all dietary fibres tested. No statistically significant differences were found among the dietary fibres in the content of oligosaccharides with the degrees of polymerisation 5 and 6 (DP 5–6). Regarding the oligosaccharides with DP 2–4, the highest contents were found in the pomegranate peel and the tomato peel dietary fibre digestions, followed by the control.

The differences in DP7 content between the different dietary fibres are clear, pointing to the differences between the dietary fibres evaluated and how the stomach acid modified them. Although there are currently no references on the production of oligosaccharides from dietary fibre after passage through the stomach, there are numerous works in which oligosaccharides are chemically and enzymatically produced from dietary fibre. Zhang et al. [58] treated citrus peel pectin and, by chemical degradation with trifluoroacetic acid, acquired three pectic oligosaccharides with molecular weight ranges (Mw) of 3000–4000 Da, 2000–3000 Da and less than 2000 Da. Additionally, Yang et al. [59] demonstrated that acid hydrolysis with lactic acid combined with an enzymatic treatment with xylanases was an effective way to produce high-purity xyloligosaccharides from poplar wood.

Regarding the oligosaccharides present after passage through the small intestine, overall, an increase in the oligosaccharide values was observed, except in the broccoli leaf dietary fibre digestion, corroborating the variability of fibre degradation depending on its mixture composition [60]. Therefore, the impact of digestion on fibre degradation and oligosaccharide production can be highly variable since several factors can influence it, such as the different types of fibre, the food matrix and the microbiological profile, among others [61]. Regarding the higher content of DP2 with respect to the previous stage, it may be due to the exposure of the substrate to mild alkali, combined with enzymatic action, which resulted in a higher yield of oligosaccharides with a lower degree of polymerisation [62]. It should also be noted that the oligosaccharides studied are resistant to the conditions of the small intestine and reach the colon available for use by the microbiota [63,64]. At the colon phase, after 8 h, the level of oligosaccharides decreased sharply to a level below 5 g/L in all dietary fibre digestions evaluated. This result could be related to the metabolic activity of the colon microbiota, which, in general, is prone to consuming oligosaccharides with a lower DP, as we have analysed [43,65]. Previous studies on simulated colon conditions have shown a similar trend [58,66,67].

### 3.4. Microbial Population Dynamics 

Table 5 shows the microbiological population dynamics during the digestion process in the small intestine and colon stages, respectively. Regarding the results of total viable bacteria, the counts increased from 2.8 log CFU/mL in the stomach to values higher than 8 log CFU/mL in the small intestine and colon. The values of total viable bacteria at the end of the small intestine ranged from 8.77 to 9.42 log CFU/mL, with the grape stems showing a significantly higher value. In contrast, no significant differences were found between the dietary fibres and stages in the colon, with values ranging from 7.9 to 8.9 log CFU/mL. The level of total viable bacteria throughout digestion was clearly associated with the counts of enterobacteria, which was the predominant microbial group. This microbial group presented counts ranging from 7.81 to 9.35 log CFU/mL and from 8 to 8.8 log CFU/mL in the small intestine and colon, respectively, with the dietary fibre samples generally showing significantly higher values than the control. Among the dietary fibres, the pomegranate peel and broccoli leaf showed the lowest significant enterobacteria counts in the small intestine and colon, respectively. The differences in dietary fibre composition could explain these results. In fact, dietary fibre constituents such as cellulose have demonstrated a positive effect on the *Enterobacteriaceae* populations in the ileum of growing pigs [68]. Regarding the colon, *Enterobacteriaceae* counts increased with the addition of dietary fibre, which is corroborated by other authors, who stated that a diet rich in dietary fibre mainly included changes in Firmicutes, Verrucomicrobia, *Enterobacteriaceae*, *Prevotella* and Bacteroides [69].

Concerning the counts of enterococci and staphylococci, both microbial groups increased from values lower than 2 log CFU/mL at the stomach level to values higher than 6 log CFU/mL during the colon stages, except for staphylococci, which significantly decreased to 3.9 log CFU/mL at the distal colon stage (Table 5). On the other hand, significant differences were found between the dietary fibres studied at the colon stage, with values ranging from 5.8 to 7.4 log CFU/mL and from 3.6 to 7.3 log CFU/mL for enterococci and staphylococci, respectively. For both microbial groups, the grape stems showed the highest mean counts in the small intestine, while the balance of these microbial groups was altered in the colon, with the tomato- and pomegranate-peel fibres showing the highest mean counts. In previous works, the growth capacities of several *Enterococcus faecium* strains were tested in the presence of dietary fibre from grape stems, which proved to be an effective substrate for promoting their growth [15].

Dietary fibre is also the main energy substrate for LAB and bifidobacteria in the colon, which have specific enzymes that break down these complex carbohydrates. In general, bifidobacteria counts, in contrast to those of LAB, were stimulated in the proximal colon in the dietary fibre samples. Specifically, the control showed significantly higher LAB levels, with 5.4 log CFU/mL, followed by the tomato and pomegranate peels, with 2.9 and 2.4 CFU/mL, respectively. In contrast, the control, with a mean value of 1.6 CFU/mL, had the lowest bifidobacteria counts (Table 5). Several studies have shown the capacity of bifidobacteria to metabolise dietary fibre from different sources [69,70], although this ability is variable among bifidobacteria strains and species. Strains of *Bifidobacterium longum, Bifidobacterium breve*, *Bifidobacterium adolescentis* and *Bifidobacterium animalis* subsp. *lactis* harbour different types of enzymes (glycosyl hydrolase) and transporters involved in the degradation of vegetable oligosaccharides and polysaccharides [71]. Thus, dietary fibres with different structures can modulate bifidobacterial growth in different ways. In our study, this fact is observed in the mean counts of bifidobacteria for the most dietary fibres studied. Concretely, the dietary fibres from grape stems, broccoli leaves and tomato peels presented mean values over 5 log CFU/mL in the colon (Table 5). Among the dietary fibres, the grape stems presented the highest mean count, with 5.4 CFU/mL. This result agrees with previous works that evaluated the bifidogenic effect of different types of fibres [25,72,73].

Regarding the yeast population, the dietary fibre samples showed significantly higher counts than the control, with the grape stem and tomato peel samples showing the highest counts, at around 3.3 log CFU/mL (Table 5). Throughout the digestion in the colon, the mean counts decreased significantly from 3.2 log CFU/mL in the proximal colon to 1.1 log CFU/mL in the distal colon. These results are in line with those obtained by other authors on fungi in the human GI tract. [74,75]. The positive effect of diary fibre on this population was evident, with higher counts in all dietary fibres with respect to the control, especially in the case of pomegranate peel (Table 6).

### 3.5. SCFA Level during Simulated Digestion

The variation in the main SCFAs found during the simulated digestion in the stomach, small intestine and colon is presented in Table 6. After stomach digestion, the mean values of acetic, propionic and butyric acids were 77.9, 23.5 and 3.9 mM, respectively. The rest of the SCFAs studied showed values lower than 0.7 mM at each stage of digestion, for all fibres evaluated. At the small intestine stage, considering that in our experiment the transfer of the digestion contents from the stomach to the small intestine involves a dilution by half, in general, the levels of SFCAs experienced a notable increase for broccoli leaf, pomegranate peel and grape-stem fibres, whereas in the case of fibres from the control and tomato peels, they were similar for acetic and butyric acids (Table 6). Finally, considering again the dilution effect during the transfer of the contents from the small intestine to the colon, in all dietary fibres assayed, acetic and butyric acids increased slightly, whereas propionic acid increased notably, up to around 10 times. These values of SFCAs were stable throughout the colon digestion stages, with mean values of 45.9, 1033 and 7.9 mM for acetic acid, propionic acid and butyric acid, respectively. Among the dietary fibres, the pomegranate-peel fibre presented the highest average acetic and butyric acid values, without significant differences with respect to the control (Table 6). As other authors have pointed out, acetic, propionic and butyric acids are the main metabolites released during the fermentation process of prebiotic compounds [76]. The sum of these metabolites is used as an indicator of fibre fermentability [77]. Based on our data, the selective growth stimulation of SFCA-producing bacteria was observed at the small intestine and colon stages, especially for propionic acid-producing bacteria, and at the lower extent for butyric acid-producing bacteria. The increase in the SFCA level in the intestine has been reported to play an important role in human health. In particular, butyric acid has been the most widely studied for its prominent effects on health; it protects against colitis and colonic cancer and displays anti-inflammatory and immunomodulatory effects [78,79]. Acetic acid is a precursor of butyric acid production and impacts lipid metabolism [80], whereas propionic acid acts as an inhibitor of gluconeogenesis and cholesterol synthesis in the liver and protects the human intestines against pathogens via its antibacterial and anti-inflammatory capacity [81].

### 3.6. Relationship of Fibre Composition, Functional Properties, SCFAs and Microbial Population to Digestion Stage and Fibre Type

**Figure 1:**
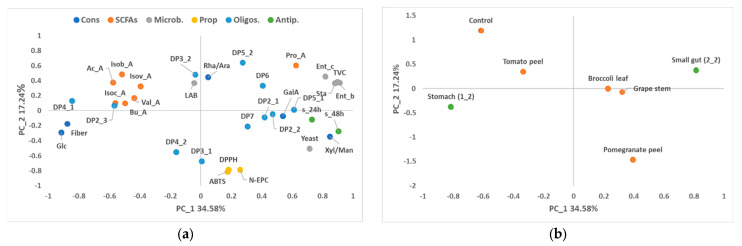
Loading plot (**a**) and score plot (**b**) after principal component analysis of dietary fibre, fibre constituents (Cons; GalA: galacturonic acid; Glc: glucose; Xyl/Gal/Man: xylose/galactose/mannose; Rha/Ara: rhamnose/arabinose), fibre properties (Prop; N-EPC: non-extractable phenolic compounds. DPPH and ABTS: antioxidant capacity), oligosaccharides (Oligos; DP: degree of polymerisation 2–7), SCFAs (acetic acid: Ac_A; propionic acid: Pro_A; butyric acid: Bu_A; isovaleric acid: Isov_A; isobutyric acid: Isob_A; isocapronic acid: Isoc_A; caproic acid: Cap_A; valeric acid: Val_A), microbial population (microb; total viable bacteria: TVC; Enterobacteria: Ent_b; Lactic acid bacteria: LAB; Enterococci: Ent_c; Staphylococci: Sta; Bifidobacteria: Bf) and antiproliferative activities (antip.) of the stomach (1_2) and small gut (2_2) samples in the planes defined by the first two principal components (PC1 and PC2).

**Figure 2:**
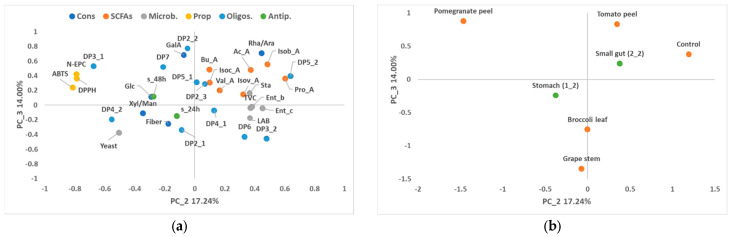
Loading plot (**a**) and score plot (**b**) after principal component analysis of dietary fibre, fibre constituents (Cons; GalA: galacturonic acid; Glc: glucose; Xyl/Gal/Man: xylose/galactose/mannose; Rha/Ara: rhamnose/arabinose), fibre properties (Prop; N-EPC: non-extractable phenolic compounds. DPPH and ABTS: antioxidant capacity), oligosaccharides (Oligos; DP: degree of polymerisation 2–7), SCFAs (acetic acid: Ac_A; propionic acid: Pro_A; butyric acid: Bu_A; isovaleric acid: Isov_A; isobutyric acid: Isob_A; isocapronic acid: Isoc_A; caproic acid: Cap_A; valeric acid: Val_A), microbial population (microb; total viable bacteria: TVC; Enterobacteria: Ent_b; Lactic acid bacteria: LAB; Enterococci: Ent_c; Staphylococci: Sta; Bifidobacteria: Bf) and antiproliferative activities (antip.) of the stomach (1_2) and small gut (2_2) samples in the planes defined by the first and third principal components (PC1 and PC3).

**Figure 3:**
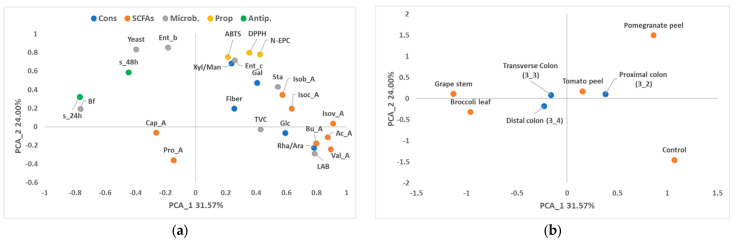
Loading plot (**a**) and score plot (**b**) after principal component analysis of dietary fibre, fibre constituents (Cons; GalA: galacturonic acid; Glc: glucose; Xyl/Gal/Man: xylose/galactose/mannose; Rha/Ara: rhamnose/arabinose), fibre properties (Prop; N-EPC: non-extractable phenolic compounds. DPPH and ABTS: antioxidant capacity), oligosaccharides (Oligos; DP: degree of polymerisation 2–7), SCFAs (acetic acid: Ac_A; propionic acid: Pro_A; butyric acid: Bu_A; isovaleric acid: Isov_A; isobutyric acid: Isob_A; isocapronic acid: Isoc_A; caproic acid: Cap_A; valeric acid: Val_A), microbial population (microb; total viable bacteria: TVC; Enterobacteria: Ent_b; Lactic acid bacteria: LAB; Enterococci: Ent_c; Staphylococci: Sta; Bifidobacteria: Bf) and antiproliferative activities (antip.) of the colon samples (proximal 3_2, transverse 3_3, and distal 3_4) in the planes defined by the two first principal components (PC1 and PC2).

**Figure 4:**
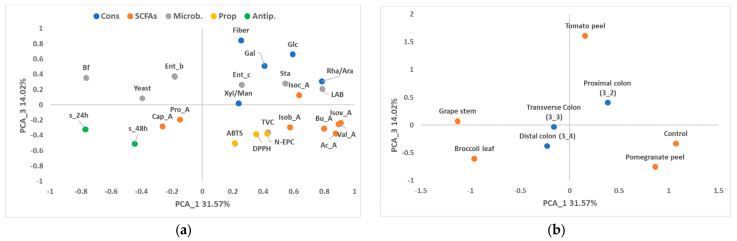
Loading plot (**a**) and score plot (**b**) after principal component analysis of dietary fibre, fibre constituents (Cons; GalA: galacturonic acid; Glc: glucose; Xyl/Gal/Man: xylose/galactose/mannose; Rha/Ara: rhamnose/arabinose), fibre properties (Prop; N-EPC: non-extractable phenolic compounds. DPPH and ABTS: antioxidant capacity), oligosaccharides (Oligos; DP: degree of polymerisation 2–7), SCFAs (acetic acid: Ac_A; propionic acid: Pro_A; butyric acid: Bu_A; isovaleric acid: Isov_A; isobutyric acid: Isob_A; isocapronic acid: Isoc_A; caproic acid: Cap_A; valeric acid: Val_A), microbial population (microb; total viable bacteria: TVC; Enterobacteria: Ent_b; Lactic acid bacteria: LAB; Enterococci: Ent_c; Staphylococci: Sta; Bifidobacteria: Bf) and antiproliferative activities (antip.) of the colon samples (proximal 3_2, transverse 3_3, and distal 3_4) in the planes defined by the first and third principal components (PC1 and PC3).

Principal component analysis (PCA) was carried out on the data for the stomach–small intestine (Figure 1 and Figure 2) and colon stages (Figure 3 and Figure 4) to obtain an interpretable overview of the main information. In the case of the PCA for stomach and small intestinal digestion, principal components 1 (PC1), 2 (PC2) and 3 (PC3) explained the variability of 34.58%, 17.24% and 14.00%, respectively (Figure 1 and Figure 2). PC1 was clearly related to the digestion stage, with the highest values for microbial counts being explained by the positive axis of PC1 and associated with samples of the small intestine (Figure 1). By contrast, samples of the stomach were on the negative axis of PC1 and associated with higher values of fibre, glucose and antiproliferative activity. The variability associated with each dietary fibre was mainly explained by PC2 and PC3 (Figure 2). High values of non-extractable phenols, antioxidant capacity and DP3_1 were associated with the pomegranate peel fibre as opposed to the control, which was linked to the production of some SCFAs, such as acetic, propanoic and isobutyric acids. On the other hand, the tomato-peel fibre was related to high values of Ram/Arb, DP2_2 and SCFAs such as acetic and isobutyric acids, in contrast to the broccoli and grape-stem fibres (Figure 1 and Figure 2).

With respect to the PCA for colon stages, the study of PC_1 (31.57% of the variability) and PC_2 (24.00% of the variability) corroborated the association of high values of non-extractable phenols and antioxidant capacity of the fibre obtained from the pomegranate peel, as well as the presence of higher amounts of most of the SCFAs in the control, together with high counts in MRS media (Figure 3 and Figure 4). In contrast to the control, broccoli and grape-stem fibres were related to low values of SCFAs, high counts of bifidobacteria and high antiproliferative activity, located on the negative axis of PC_1. PC_3 (14.02% of the variability) was mainly associated with the variability of tomato-peel fibre, which was related to the highest values of fibre (Figure 4).

In the case of the colon samples, the factor “stage” had a low influence on the global variability (Figure 3 and Figure 4). However, it can be observed that the samples obtained from the proximal part of the colon are associated with higher values of dietary fibre and some of its constituents, such as glucose (Glc) and rhamnose/arabinose (Rha/Ara). Additionally, this initial stage of the colon showed higher counts of lactic acid bacteria (LAB), Enterococci, (Ent_c), and staphylococci (Sta).

## 4. Conclusions

In conclusion, the source and composition of dietary fibre play a crucial role in determining its functional properties and behaviour during intestinal transit. Notably, dietary fibre derived from pomegranate peel maintains its high phenol content throughout the entire process of digestion simulation in the colon. This persistence of phenols may enhance intestinal health, owing to its heightened antioxidant activity. Similarly, dietary fibre sourced from broccoli and grape stems exhibits anti-proliferative activity within both the small intestine and the colon while also leading to significant modification of the colonic microbiology. This type of dietary fibre favours the proliferation of bifidobacteria, as opposed to LAB, and high amounts of SCFAs. However, for a more comprehensive understanding of the impact of various types of dietary fibre on the microbial community comprising the colonic microbiota, it is imperative to identify, quantify and monitor them using high-throughput sequencing techniques in future research endeavours.

## Figures and Tables

**Table 1:** Simulated digestion program in a three-bioreactor BIOSTAT A system: experimental conditions, reactive concentrations and sampling description.

*Stage*	Conditions					Operation		
Time	Vol (mL)	Temp (°C)	pH ^1^	pO ^2^	Stir (rpm)	Type	Vol (mL)	Description
***Digestion***								
0:00:00	600	20	6–6.5	21	---	Sampling	−100	Initial sample
0:10:00	0					Transfer to	500	Stomach
***Stomach (bioreactor 1)***								
0:00:00	750	37	2.5	<0.5	150	Initial content	750	PBS
0:10:00	1350					Transfer from	500	Batch
						Supplementation	100	Pepsin 4.5%
						Sampling 1_1	−25	Microbial control
3:00:00	1225					***Sampling 1_2***	−100	Stomach sample
3:15:00	500					Transfer to	725	Small intestine
***Small intestine (bioreactor 2)***								
0:00:00	750	37	6.5	<0.5	150	Initial content	750	PBS
3:15:00	1475					Transfer from	750	Stomach
						Supplementation	100	Pancreatin (3%); Bilis (7.5%)
						Sampling 2_1	−25	Microbial control
7:00:00			6.5			Start pH grad. ^3^		
11:00:00	1450		7			End pH grad.		
						***Sampling 2_2***	−100	Small intestine sample
11:15:00	500					Transfer to	950	Large gut
***Colon (bioreactor 3)***								
0:00:00	750	37	5.7	<0.5	150	Initial content	750	PBS
0:05:00	770					Supplementation	25	MRS
0:10:00	790					Supplementation	15	Faecal inoculum
11:00:00	750					Sampling 3_1	−40	Microbial control
11:15:00	1700					Transfer from	1000	Small intestine
			5.7			Start pH grad.		
24:00:00	1600					***Sampling 3_2***	−100	Ascending sample
31:00:00			6			pH grad. point		
48:00:00	1500					***Sampling 3_3***	−100	Transverse sample
50:00:00			6.4			pH grad. point		
70:00:00			6.8			End pH grad.		
72:00:00	1400					***Sampling 3_4***	−100	Descending sample

^1^ Manual adjustment of the initial pH with HCl 5 M and Na_2_CO_3_ 2 M; automatic pH adjustment during the digestion process with HCl 0.5 M and Na_2_CO_3_ 0.2 M. ^2^ Oxygen pressure adjusted with nitrogen: CO_2_ (99.5:0.5). ^3^ Grad.: gradient.

**Table 2:** Constituents and properties of feed supplemented with different types of dietary fibre prior to digestion.

Parameters ^2^		Control	Dietary Fibre Type ^1^				*p*-Values
			B_Leaf	P_Peel	G_Stem	T_Peel	
	Dietary fibre (g/kg)	227	226	202	223	188	0.703
Fiber constituents (mg/g dietary fibre)							
	GalA	578	588	686	669	526	0.121
	Glc	34.3 ^b,3^	27.2 ^ab^	35.5 ^b^	25.6 ^a^	34.2 ^b^	0.035
	Xyl/Gal/Man	<0.5 ^a^	2.12 ^b^	2.43 ^b^	2.21 ^b^	<0.5 ^a^	<0.001
	Rha/Ara	3.3	2.3	2.4	1.9	2.8	0.310
Fiber properties							
	N-EPC (mg GAE/100 g)	1.6 ^a^	3.7 ^a^	83.5 ^c^	15.7 ^b^	2.2 ^a^	<0.001
	ABTS (mg Trolox/100 g)	229 ^abc^	261 ^abc^	175 ^a^	266 ^bc^	216 ^ab^	0.025
	DPPH (mg Trolox/100 g)	70 ^abc^	55 ^ab^	621 ^d^	95 ^bc^	23 ^a^	<0.001
	Antiprol (% g inh)	100.0	80.4	103.2	85.3	93.1	0.120

^1^ B_leaf: broccoli leaf; P_peel: pomegranate peel; G_stem: grape stem; T_peel: tomato peel. ^2^ GalA: galacturonic acid; Glc: glucose; Xyl/Gal/Man: xylose/galactose/mannose; Rha/Ara: rhamnose/arabinose; N-EPC: non-extractable phenolic compounds. DPPH and ABTS: antioxidant capacity; Antiprol (% g inh): antiproliferative activity (% growth inhibition). ^3^ Mean values with different superscript letters (^abcd^) are significantly different (*p* ≤ 0.05) among fibre samples.

**Table 3:** Amount, composition and functional properties of dietary fibre after stomach stage (sampling 1_2), small intestine stage (sampling 2_1) and colon stages (proximal (prox): sampling 3_2; transverse (trans): sampling 3_3; distal: sampling 3_4).

Parameters ^2^			Control	Dietary Fibre ^1^						
				B_Leaf ^1^	P_Peel	G_Stem	T_Peel			
***Stomach simulation***										
	Dietary Fibre (g/kg)		172.7 ^b,3^	127.6 ^a^	123.7 ^a^	163.9 ^b^	155.3 ^b^			
	*Fiber constituents (mg/g dietary fibre)*									
		GalA	577.9	587.9	686.1	669.3	526.2			
		Glc	34.3 ^bc^	27.2 ^ab^	35.5 ^c^	25.6 ^a^	34.2 ^bc^			
		Xyl/Gal/Man	<0.5 ^a^	2.1 ^b^	2.4 ^b^	2.2 ^b^	<0.5 ^a^			
		Rha/Ara	3.3	2.3	2.4	1.9	2.8			
	*Fiber properties*									
		N-EPC (mg GAE/100 g)	0.79 ^a^	1.99 ^a^	62.31 ^c^	12.89 ^b^	1.68 ^a^			
		ABTS (mg Trolox/100 g)	216 ^ab^	387 ^c^	933 ^d^	387 ^bc^	291 ^b^			
		DPPH (mg Trolox/100 g)	111.1 ^a^	62.7 ^a^	636.4 ^c^	235.1 ^b^	90.7 ^a^			
		Antiprol (% g inh)	105	80	105	88	92			
***Small intestine simulation***										
	Fiber (g/kg)		70.3 ^b^	58.8 ^a^	65.3 ^b^	85.6 ^c^	89.7 ^c^			
	*Fiber constituents (mg/g dietary fiber)*									
		GalA	626.8	613.1	699.3	565.0	708.4			
		Glc	22.6	18.8	21.9	18.1	23.6			
		Xyl/Gal/Man	2.6 ^a^	3.9 ^ab^	4.0 ^b^	2.9 ^ab^	2.8 ^ab^			
		Rha/Ara	8.9 ^b^	4.9 ^a^	6.5 ^ab^	3.9 ^a^	5.5 ^ab^			
	*Fiber properties*									
		N-EPC (mg GAE/100 g)	1.24 ^a^	1.23 ^a^	75.81 ^b^	6.25 ^a^	4.25 ^a^			
		ABTS (mg Trolox/100 g)	356 ^a^	310 ^a^	708 ^b^	351 ^a^	246 ^a^			
		DPPH (mg Trolox/100 g)	27.0 ^a^	3.2 ^a^	860.6 ^c^	149.0 ^b^	2.8 ^a^			
		Antiprol (% g inh)	99.0 ^b^	76.5 ^a^	55.6 ^a^	56.9 ^a^	82.2 ^b^	Stage		
***Colon simulation***								Prox	Trans	Distal
	Fiber (g/kg)		59 ^b^	46 ^a^	59 ^b^	66 ^b^	76 ^c^	69 ^B,4^	59 ^A^	56 ^A^
	*Fiber constituents (mg/g dietary fibre)*									
		GalA	600.3	593.0	659.8	589.4	690.6	626.8	625.4	627.7
		Glc	20.8 ^c^	13.2 ^ab^	17.8 ^bc^	11.8 ^a^	26.6 ^d^	19.3 ^B^	19.1 ^AB^	15.8 ^A^
		Xyl/Gal/Man	2.4 ^a^	3.0 ^ab^	3.9 ^c^	2.5 ^ab^	3.2 ^b^	3.1	3.1	2.8
		Rha/Ara	8.5 ^d^	3.0 ^b^	5.7 ^c^	1.3 ^a^	7.0 ^cd^	5.4 ^B^	5.8 ^B^	4.0 ^A^
	*Fiber properties*									
		N-EPC (mg GAE/100 g)	3.7 ^a^	2.3 ^a^	39.3 ^c^	6.3 ^b^	3.2 ^a^	11.1 ^B^	13.0 ^C^	8.8 ^A^
		ABTS (mg Trolox/100 g)	302 ^ab^	345 ^b^	732 ^d^	436 ^c^	274 ^a^	405 ^A^	447 ^B^	402 ^A^
		DPPH (mg Trolox/100 g)	20 ^a^	22 ^a^	534 ^c^	99 ^b^	16 ^a^	141 ^B^	160 ^B^	115 ^A^
		Antiprol (% g inh)	103 ^c^	80 ^ab^	89 ^abc^	77 ^a^	97 ^bc^	96 ^B^	80 ^A^	92 ^AB^

^1^ B_leaf: broccoli leaf; P_peel: pomegranate peel; G_stem: grape stem; T_peel: tomato peel. ^2^ Mean values with different superscript letters (^abcd^) are significantly different (*p* ≤ 0.05) among dietary fibre samples. ^3^ GalA: galacturonic acid; Glc: glucose; Xyl/Gal/Man: xylose/galactose/mannose; Rha/Ara: rhamnose/arabinose; N-EPC: non-extractable phenolic compounds. DPPH and ABTS: antioxidant capacity; Antiprol (% g inh): antiproliferative activity (% growth inhibition). ^4^ Mean values with different superscript capital letters (^ABC^) are significantly different (*p* ≤ 0.05) among colon stage samples.

**Table 4:** Oligosaccharides concentration (g/L) after stomach stage (sampling 1_2) and small intestine stage (sampling 2_1).

Oligosaccharides		Control	Dietary Fibre ^1^			
			B_Leaf	P_Peel	G_Stem	T_Peel
***Stomach simulation***						
	DP7	6.42 ^b,2^	2.29 ^a^	23.60 ^e^	11.73 ^c^	19.99 ^d^
	DP6	0.04	<0.01	<0.01	0.03	<0.01
	DP5 1	<0.01	0.04	0.02	0.08	0.05
	DP5 2	0.10	0.04	0.08	<0.01	0.06
	DP4 1	0.95 ^b^	0.56 ^a^	0.20 ^a^	0.92 ^b^	0.86 ^b^
	DP4 2	<0.01 ^a^	0.67 ^b^	0.66 ^b^	1.16 ^d^	1.02 ^c^
	DP3 1	1.72 ^b^	1.01 ^a^	2.78 ^c^	1.05 ^a^	1.08 ^a^
	DP3 2	0.93 ^c^	<0.01 ^a^	<0.01 ^a^	0.85 ^b^	<0.01 ^a^
	DP2 1	1.14 ^b^	1.07 ^a^	4.54 ^d^	1.76 ^c^	1.10 ^ab^
	DP2 2	1.60 ^b^	2.57 ^b^	6.83 ^b^	<0.01 ^a^	2.07 ^b^
	DP2 3	<0.01 ^a^	<0.01 ^a^	<0.01 ^a^	<0.01 ^a^	4.64 ^b^
***Small intestine simulation***						
	DP7	18.59 ^b^	1.74 ^a^	25.50 ^d^	22.68 ^c^	22.14 ^c^
	DP6	0.28 ^b^	<0.01 ^a^	<0.01 ^a^	1.05 ^c^	<0.01 ^a^
	DP5 1	<0.01 ^a^	<0.01 ^a^	0.59 ^b^	0.47 ^b^	1.18 ^c^
	DP5 2	0.79 ^d^	0.26 ^b^	<0.01 ^a^	<0.01 ^a^	0.67 ^c^
	DP4 1	0.52 ^d^	<0.01 ^a^	0.26 ^c^	0.11 ^b^	0.22 ^c^
	DP4 2	<0.01 ^a^	0.44 ^b^	0.54 ^b^	0.43 ^b^	0.69 ^c^
	DP3 1	1.17 ^c^	0.90 ^a^	2.73 ^d^	0.88 ^a^	1.07 ^b^
	DP3 2	0.90 ^b^	<0.01 ^a^	<0.01 ^a^	1.23 ^c^	<0.01 ^a^
	DP2 1	1.84 ^c^	<0.01 ^a^	3.37 ^d^	12.36 ^e^	1.07 ^b^
	DP2 2	13.06 ^b^	<0.01 ^a^	19.28 ^d^	<0.01 ^a^	17.37 ^c^

^1^ B_leaf: broccoli leaf; P_peel: pomegranate peel; G_stem: grape stem; T_peel: tomato peel. ^2^ Mean values with different superscript letters (^abcde^) are significantly different (*p* ≤ 0.05) among dietary fibre samples.

**Table 5:** Mean microbial counts (log CFU/mL) after small intestine stage (sampling 2_1) and colon stages (proximal (prox): sampling 3_2; transverse (trans): sampling 3_3; distal: sampling 3_4).

Microbial Population		Control	Dietary Fibre ^1^						
			B_Leaf	P_Peel	G_Stem	T_Peel			
***Small intestine simulation***									
	Total viable bacteria	8.91 ^b,2^	9.18 ^c^	7.81 ^a^	9.42 ^d^	8.77 ^b^			
	Enterobacteria	8.54 ^b^	9.35 ^c^	7.81 ^a^	9.22 ^c^	9.00 ^c^			
	Lactic acid bacteria	5.18 ^b^	<1 ^a^	<1 ^a^	<1 ^a^	<1 ^a^			
	Enterococci	4.19 ^ab^	5.93 ^bc^	3.00 ^a^	7.39 ^c^	4.94 ^ab^			
	Staphylococci	4.95 ^a^	6.11 ^b^	5.76 ^b^	7.47 ^c^	7.14 ^c^			
	Yeast	1.2 ^a^	2.65 ^b^	2.93 ^b^	3.85 ^c^	3.33 ^c^	Stage		
***Colon simulation***							Prox.	Trans.	Distal
	Total viable bacteria	8.9	8.6	9.0	7.9	8.7	8.4	8.8	8.7
	Enterobacteria	8.0 ^a^	8.4 ^b^	8.8 ^c^	8.7 ^c^	8.8 ^c^	8.6 ^B,3^	8.6 ^B^	8.4 ^A^
	Lactic acid bacteria	5.4 ^d^	<1 ^a^	2.4 ^b^	<1 ^a^	2.9 ^c^	3.2 ^C^	2.2 ^B^	1.1 ^A^
	Enterococci	5.8 ^a^	6.4 ^b^	7.5 ^c^	5.8 ^a^	7.4 ^c^	6.7	6.5	6.5
	Staphylococci	5.3 ^c^	3.9 ^b^	7.3 ^d^	3.6 ^a^	7.0 ^d^	6.0 ^B,3^	6.4 ^C^	3.9 ^A^
	Bifidobacteria	1.6 ^a^	5.1 ^c^	2.8 ^b^	5.4 ^d^	5.2 ^cd^	3.2 ^A^	4.9 ^C^	3.9 ^B^
	Yeast	1.1 ^a^	2.2 ^b^	2.5 ^b^	3.5 ^c^	3.1 ^c^	3.2 ^C^	2.2 B	1.1 A

^1^ B_leaf: broccoli leaf; P_peel: pomegranate peel; G_stem: grape stem; T_peel: tomato peel. ^2^ Mean values with different superscript letters (^abcd^) are significantly different (*p* ≤ 0.05) among dietary fibre samples. ^3^ Mean values with different superscript capital letters (^ABC^) are significantly different (*p* ≤ 0.05) among colon stage samples.

**Table 6:** SCFA concentrations (mM) after stomach stage (sampling 1_2), small intestine stage (sampling 2_1) and colon stages (proximal (prox): sampling 3_2; transverse (trans): sampling 3_3; distal: sampling 3_4).

SCFAs		Control	Dietary Fibre ^1^						
			B_Leaf	P_Peel	G_Stem	T_Peel			
***Stomach simulation***									
	Acetic acid	191.08 ^c,2^	20.44 ^a^	60.17 ^ab^	18.51 ^a^	99.36 ^b^			
	Propionic acid	34 ^b^	32 ^b^	33 ^b^	33 ^b^	24 ^a^			
	Butyric acid	5.49 ^c^	2.09 ^a^	3.74 ^b^	1.77 ^a^	6.42 ^c^			
	Isovaleric acid	0.63 ^b^	0.08 ^a^	0.19 ^a^	0.06 ^a^	0.05 ^a^			
	Isobutyric acid	0.03	<0.01	<0.01	<0.01	0.08			
	Isocaproic acid	<0.01	<0.01	<0.01	<0.01	1.22			
***Small intestine simulation***									
	Acetic acid	104.62 ^b^	36.83 ^a^	65.31 ^ab^	24.03 ^a^	50.10 ^a^			
	Propionic acid	60	59	60	60	51			
	Butyric acid	2.34 ^ab^	5.33 ^b^	3.30 ^ab^	1.80 ^a^	3.80 ^ab^			
	Isovaleric acid	0.31	0.02	0.13	0.14	0.07			
	Isobutiric acid	0.05	0.02	0.02	<0.01	0.02			
	Isocaproic acid	0.06	<0.01	<0.01	<0.01	0.02			
	Valeric acid	0.17	0.01	0.11	0.03	0.11	Stage		
***Colon simulation***							Prox.	Trans.	Distal
	Acetic acid	82.1 ^b^	27.6 ^a^	70.8 ^b^	17.6 ^a^	31.6 ^a^	47.7	44.7	45.4
	Propionic acid	873	712	1033	1194	1354	1006	1033	1060
	Butyric acid	13.0 ^c^	5.4 ^a^	10.4 ^bc^	3.8 ^a^	7.1 ^ab^	7.8	7.7	8.4
	Isovaleric acid	<0.01	<0.01	<0.01	<0.01	<0.01	<0.01	<0.01	<0.01
	Isobutyric acid	<0.01	<0.01	<0.01	<0.01	<0.01	<0.01	<0.01	<0.01
	Isocaproic acid	<0.01	<0.01	<0.01	<0.01	<0.01	<0.01	<0.01	<0.01
	Caproic acid	<0.01	<0.01	<0.01	<0.01	<0.01	<0.01	<0.01	<0.01
	Valeric acid	<0.01	<0.01	<0.01	<0.01	<0.01	<0.01	<0.01	<0.01

^1^ B_leaf: broccoli leaf; P_peel: pomegranate peel; G_stem: grape stem; T_peel: tomato peel. ^2^ Mean values with different superscript letters (^abc^) are significantly different (*p* ≤ 0.05) among dietary fibre samples.

## Data Availability

The original contributions presented in the study are included in the article, further inquiries can be directed to the corresponding author.
